# Electron microscopy analysis of astrocyte-synapse interactions shows altered dynamics in an Alzheimer’s disease mouse model

**DOI:** 10.3389/fncel.2023.1085690

**Published:** 2023-01-26

**Authors:** Mandy S. J. Kater, Aina Badia-Soteras, Jan R. T. van Weering, August B. Smit, Mark H. G. Verheijen

**Affiliations:** ^1^Department of Molecular and Cellular Neurobiology, Center for Neurogenomics and Cognitive Research, Amsterdam Neuroscience, Vrije Universiteit Amsterdam, Amsterdam, Netherlands; ^2^Department of Human Genetics, Center for Neurogenomics and Cognitive Research, Amsterdam Neuroscience, Amsterdam University Medical Center, Vrije Universiteit Medical Center, Amsterdam, Netherlands

**Keywords:** glia, tripartite synapse, perisynaptic astrocytic processes, leaflet, synapse, memory consolidation, APP/PS1

## Abstract

**Introduction:**

Astrocyte-synapse bi-directional communication is required for neuronal development and synaptic plasticity. Astrocytes structurally interact with synapses using their distal processes also known as leaflets or perisynaptic astrocytic processes (PAPs). We recently showed that these PAPs are retracted from hippocampal synapses, and involved in the consolidation of fear memory. However, whether astrocytic synaptic coverage is affected when memory is impaired is unknown.

**Methods:**

Here, we describe in detail an electron microscopy method that makes use of a large number of 2D images to investigate structural astrocyte-synapse interaction in paraformaldehyde fixed brain tissue of mice.

**Results and discussion:**

We show that fear memory-induced synaptic activation reduces the interaction between the PAPs and the presynapse, but not the postsynapse, accompanied by retraction of the PAP tip from the synaptic cleft. Interestingly, this retraction is absent in the APP/PS1 mouse model of Alzheimer’s disease, supporting the concept that alterations in astrocyte-synapse coverage contribute to memory processing.

## 1. Introduction

Astrocytes are specialized cells in the mammalian brain and are implicated in a variety of metabolic, homeostatic, and structural functions. They display well-delineated busy territories that consist of several primary, secondary and higher order processes that account for 70–80% of the astrocyte territory (Khakh and Sofroniew, 2015). The most distal fine processes contact other astrocytes, blood vessel or neurons (Santello et al., 2019). These fine processes, also known as leaflets or perisynaptic astrocytic processes (PAPs), interact with synapses (Ventura and Harris, 1999; Khakh and Sofroniew, 2015; Ghézali et al., 2016). This structure, identified as the tripartite synapse, is involved in the regulation of neuronal activity and synaptic transmission (Araque et al., 1999; Semyanov and Verkhratsky, 2021; Saint-Martin and Goda, 2022). The first evidence supporting the role of astrocytes at the synapse came from observations that astrocytes sense neuronal activity and respond by elevating the intracellular concentration of Ca^2+^, consequently leading to the release of gliotransmitters such as glutamate, ATP, D-serine and lactate that in turn modulate synaptic activity (Araque et al., 1999; Bezzi and Volterra, 2001; Dallérac et al., 2013; Sancho et al., 2021). PAPs are motile and show a retraction from the synapse in response to synaptic activity, subsequently leading to regulation of levels of neurotransmitters followed by modulation of the functional outcome of local transmission (Hirrlinger et al., 2004; Benediktsson et al., 2005; Araque et al., 2014; Perez-Alvarez et al., 2014; Hillen et al., 2018; Henneberger et al., 2020). However, defective PAP motility could lead to a continuous excess of, for instance, extracellular glutamate leading to neuronal hyperexcitation and subsequent neuronal death (Mahmoud et al., 2019). Thus, the spatial and in particular dynamic functional interaction between PAPs and synapses is important for optimal brain functioning.

There has been an increasing interest in the role of astrocytes on synaptic activity in memory processes. Recently, our group has demonstrated that fear memory consolidation induces a transient retraction of the hippocampal astrocytic processes from the synaptic cleft, a process that was found to enhance the strength of fear memory expression (Badia-Soteras et al., 2022). Fear memory recall is impaired in neurodegenerative disorders such as Alzheimer’s disease (AD). In APP/PS1 mice, a commonly used mouse model of AD in which amyloidosis is mimicked, weakening of synapses and reactive astrogliosis are described (Acosta et al., 2017; Arranz and De Strooper, 2019; Preman et al., 2021; Hulshof et al., 2022). Pathological reactive astrocytes display a hypertrophy of their processes (Rose et al., 1976), which may hamper the interaction between PAPs and synapses. Whether astroglial synapse coverage is affected and subsequently leads to impaired memory recall in AD mice remains to be determined.

Transmission electron microscopy (EM) is an imaging technique that enables the ability to visualize the ultrastructure of the tripartite synapse (Tremblay et al., 2010). In comparison to other imaging techniques, such as confocal microscopy and two-photon imaging, EM displays a superior spatial resolution which allows to obtain quantitative information measured at nanoscale, e.g., synapse and astrocyte surface, synaptic density and cell organelles (Harris and Weinberg, 2012; Kuipers et al., 2016; Courtland, 2018; Kubota et al., 2018). Moreover, the distance between cells can be visualized with high accuracy and is therefore beneficial to study the spatial interaction between structures.

In this study, we report in detail a method to study the spatial alterations of the tripartite synapse by 2D EM analysis, which allows the inclusion of a high number of observations per animal realizing a highly reliable dataset. We stimulated synaptic activity by using a contextual fear conditioning paradigm and fixed the brain tissue with paraformaldehyde. Here, we observed that fear memory-induced retraction of PAPs is impaired in the APP/PS1 mouse model.

## 2. Materials and methods

### 2.1. Animals

All experimental procedures were approved by the local animal research committee and complied with the European Council Directive (86/609/EEC). APP/PS1 mice (The Jackson Laboratory, Bar Harbor, ME, USA; strain B6C3-Tg (APPswe, PSEN1dE9) 85Dbo/J; stock number 004462) express a chimeric mouse/human APP gene harboring the Swedish double mutation K595N/M596L (APPswe) and a human PS1 gene harboring the exon 9 deletion (PS1dE9), both under control of the mouse prion protein promotor (MoPrp.Xho) (Jankowsky et al., 2004). These mice were kept on a C57BL6/J genetic background. APP/PS1 mice and wild-type (WT) littermates were used for EM analysis at 4 months of age. The mice were individually housed in Macrolon cages on sawdust bedding with a 12 h light/dark cycle (7 a.m. lights on; 7 p.m. lights off) and *ad libitum* access to food and water. Housing was controlled for temperature and humidity.

### 2.2. Contextual fear conditioning

Fear conditioning was performed in the morning of the light phase. All mice were handled for two consecutive days prior to the experiment to prevent stress induced by the researcher. The mice that were used as sample for EM experiments were placed in a conditioning chamber (TSE systems, Berlin, Germany), consisting of Plexiglas with a stainless steel grid floor. Illumination (100–500 lx) and background sound (68 dB, white noise) were controlled. After 120 s of exploration, the mice received a 2 s 0.7 mA foot-shock. They remained in the conditioning chamber for 30 s before return to the home cage. The chamber was cleaned with 70% ethanol in between sessions. Home-caged (HC) mice were not exposed to the conditioning chamber.

For the testing of fear memory, 3 month old WT and APP/PS1 mice (*n* = 12 per group) underwent fear conditioning in a conditioning chamber that consisted of a transparent acrylic box with a floor of stainless steel rods connected to a shock generator (25 cm × 18 cm × 21 cm; San Diego Instruments, San Diego, USA). Mice were allowed to explore the context for 2 min, followed by a total of three foot shocks (0.7 mA, 2 s) with a 30 s interval. The retrieval test was performed 24 h following conditioning. Mouse movement was video recorded at a frame rate of 30 frames per second. Freezing behavior was measured when a mouse did not move, except for respiratory activity, for a minimum of 30 frames. In between trials the cages were cleaned with 70% ethanol.

### 2.3. Sample preparation

Four hours after conditioning, mice (*n* = 5 WT and *n* = 5 APP/PS1) were sacrificed by transcardial perfusion with ice-cold 4% paraformaldehyde (PFA) depolymerized in phosphate buffered saline (PBS, 137 mM NaCl, 2.7 mM KCl, 10 mM Na_2_HPO_4_, 1.9 mM KH_2_PO_4_, pH = 7.4) under anesthesia by 250 mg/kg Avertine. PFA perfusion was preceded with PBS (30 s) and the flow of the pumps was set at 15 ml/min for 6 min. Home cage mice (*n* = 5 WT and *n* = 5 APP/PS1) were perfused by an identifical protocol. Whole brain was dissected and kept in 4% PFA for 24 h after which the solution was replaced for 30% sucrose in PBS to cryopreserve the tissue. Next, the fixed brain tissue was frozen by storage at −80°C until further processed. Fifty μm thin coronal sections of the hippocampus were made on the cryostat and kept in PBS for a maximum of 24 h. Free-floating sections were post-fixed with 1% osmium and 1% ruthenium and subsequently exposed to increasing ethanol concentrations (30, 50, 70, 90, 96, and 100%) and propylene oxide for dehydration. Subsequently, sections were embedded in epoxy resin and polymerized for 72 h at 65°C. Ultra-thin sections of 90 nm of the dorsal CA1 hippocampus were cut on an ultra-microtome (Reichert Jung Ultracut E, London, UK) and collected on 300-mesh copper grids. Finally, the sections were contrasted with uranyl acetate and lead citrate in an ultra-stainer (Leica Microsystems, EM AC20, Wetzlar, Germany).

### 2.4. Electron microscopy imaging and analysis

The grids were examined with a JEOL JEM 1011 EM (JEOL, Akishima, Tokyo, Japan) at 50.000× magnification. Pictures were taken with an side-mounted Moreda 11-MP camera (EMSIS GmbH, Münster, Germany) and iTEM software (Olympus IMS, Waltham, MA, USA). A total of 100 pictures of randomly selected locations within the dorsal CA1 hippocampus (*stratum radiatum*) were collected in which a total of 130 morphologically intact synapses with a recognizable pre- and post-synaptic area [containing synaptic vesicles and a post-synaptic density (PSD), respectively] were identified and used for analysis. Astrocytes were identified by a relatively clear cytoplasm, stellate morphology, absence of neurofilaments and presence of glycogen granules. As five animals per condition were included, this yielded the analysis of a total of 650 synapses per condition. Analysis was performed in ImageJ 1.53f51 (National Institutes of Health, Bethesda, MD, USA) were the pre-synapse and post-synapse perimeter of each synapse were measured. In addition, the number of synapses contacting a PAP was determined. When in contact with a PAP, the contact site between synapse and astrocyte was measured. When the astrocyte PAP was contacting the synaptic cleft, the distance between astrocyte PAP and PSD and the astrocyte PAP tip length were measured. Synapses that seemed to have multiple PSDs were excluded from analysis. The observer was blinded for genotype and experimental condition until statistical analysis.

### 2.5. Statistical analysis

GraphPad Prism 9.4.1 (GraphPad Software, La Jolla, CA, USA) was used for graphical representation and statistical analysis of the data. The number of animals used for each analysis (*n*) as well as the number of included synapses (*N*) are indicated in the figure legends as *n*/*N*. All figures show means ± standard error of the mean (SEM). Individual data points are averaged values per sample. Outlier removal was performed using the ROUT method (*Q* = 1%). Normality was tested with the Kolmogorov–Smirnov test. The data was log-transformed to realize a normal distribution when required. Statistical comparisons were based on the data obtained from multiple unique synapses measured within several animals, and therefore a multi-level regression analysis was performed (Aarts et al., 2014). Thus, statistical differences between conditions was determined by a nested *t*-test. For comparison of more than two groups, a nested one-way ANOVA was used. A chi-square test followed by Fisher’s exact test was used on categorical data and was performed in RStudio 1.3.1093. Recommended *post-hoc* tests were applied. The level of significance was set at *P* ≤ 0.05. All statistical details are reported in Supplementary Table 1.

## 3. Results

### 3.1. Measuring the synaptic ultrastructure and PAP-synapse interactions in the CA1 hippocampus of wild-type mice

**FIGURE 1:**
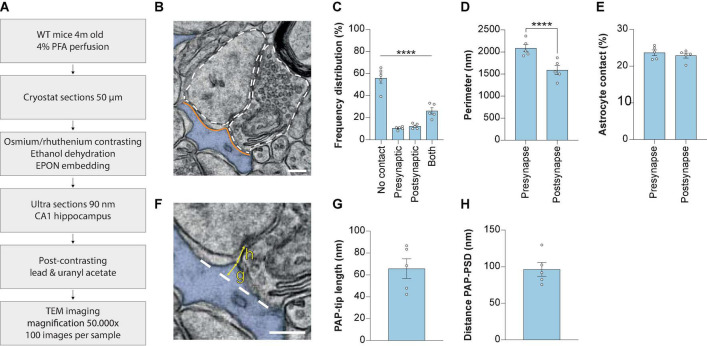
Measuring synaptic ultrastructure and astrocyte interactions by 2D EM analysis in mouse hippocampus CA1. **(A)** Schematic illustration of the methods used. **(B)** Example 2D EM image of a synapse in mouse hippocampus CA1. The outline of the pre-synaptic and post-synaptic membranes is indicated with a dashed line. The astrocyte is pseudo-colored in blue. Orange lines indicate the membrane interaction between pre-synapse and post-synapse with the astrocyte. **(C)** Frequency distribution plot showing the percentages of presence of contact types between synapse and astrocyte. **(D)** Perimeter of the pre-synapse and post-synapse. **(E)** Contact between astrocyte and pre-synapse or post-synapse, relative to the pre-synapse or post-synapse perimeter, respectively. **(F)** Example of measurement of PAP tip length **(G)** and distance between the PAP-tip and PSD **(H)**. The base of the protruding part of the PAP-tip is indicated with the dashed line. **(G)** Quantification of the PAP-tip to PSD distance. **(H)** Quantification of the astrocyte PAP tip length. Data is presented as mean SEM. ***P≤ 0.0001. PAP, perisynaptic astrocytic process; PSD, post-synaptic density. Scale bars are 300 nm *n* = 5/441. Statistical details are reported in Supplementary Table 1.

The spatial interactions between excitatory synapses and astrocytes in the *stratum radiatum* of the CA1 region of the hippocampus were studied in detail using EM (Figures 1A, B). First, for each synapse we determined, based on the 2D image, whether there was membrane contact with an astrocyte. If there was contact, we determined whether the interaction occurred at the pre-synapse site only, at the post-synapse site only or both (Figure 1C). Most synapses showed no contact with astrocytes (54.03 ± 4.23%) and only a fraction of the synapses had solely presynaptic contact (9.83 ± 0.83%) or only post-synaptic contact (11.53 ± 1.15%). Contact with both presynaptic and post-synaptic elements was observed more frequently (24.62 ± 3.14%). Thus, in line with previous EM data (Ventura and Harris, 1999), we show that the majority of excitatory synapses in the *stratum radiatum* of the hippocampus CA1 measured by a 2D-analysis are not contacted by an astrocyte, and that the type of contact varies significantly.

Next, the perimeters of the pre-synapse and post-synapse were measured (Figure 1B, white dashed lines) and revealed that CA1 hippocampal pre-synapses have a larger perimeter than post-synapses (Figure 1D). The extent of astrocytic contact with either pre-synapse or post-synapse was measured by taking the length of the shared membrane (Figure 1B, orange lines). We found a shorter physical contact between the astrocyte and the post-synaptic membrane in comparison to the pre-synaptic membrane (Supplementary Figure 1). Subsequently, we calculated the PAP contact relative to the pre-synapse or post-synapse perimeter and observed no significant differences between the extent of pre-synapse/astrocyte and post-synapse/astrocyte contact (Figure 1E).

A more detailed analysis of astrocyte-synapse interaction was performed by focusing at the PAP tip interacting with the synaptic cleft. The length of the PAP tip, indicating how far the PAP was extending from the PAP base (Figure 1F, yellow arrow indicated with “g”) was 65.8 nm ± 8.3 (Mean ± SEM; Figure 1G). A second parameter we measured was the PAP-PSD distance, which is the shortest distance between the PAP tip protruding toward the synaptic cleft and the edge of the PSD (Figure 1F, yellow arrow indicated with “h”), and we found this distance was 96.4 nm ± 8.6 (Mean ± SEM; Figure 1H). Thus, using a 2D EM analysis performed in fixed mouse brain slices enables the opportunity to study structural properties of CA1 excitatory synapses.

### 3.2. The structural properties of excitatory synapses are unaffected by delayed shock in WT and APP/PS1 mice

**FIGURE 2:**
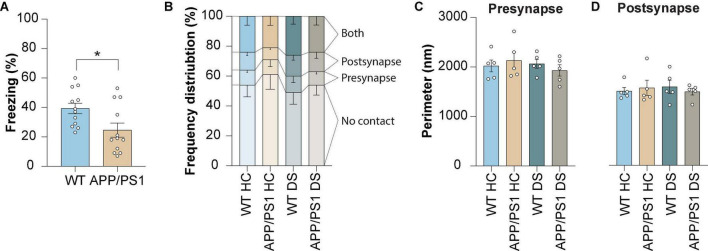
Contextual memory formation induced by delayed shock in WT and APP/PS1 mice does not affect the proportion of astrocyte-synapse contact or synapse size. **(A)** Freezing levels measured during contextual fear conditioning in WT and APP/PS1 mice at 3 months of age. **(B)** Frequency distribution plot of contact categories at 4 months of age. **(C)** Pre-synapse perimeter at 4 months of age. **(D)** Post-synapse perimeter at 4 months of age. Data is presented as mean ± SEM. *P = ≤ 0.05. **(A)** WT *n* = 12, APP/PS1 *n* = 12; **(B)** WT HC *n* = 5/441, APP/PS1 HC *n* = 5/376, WT DS *n* = 431, APP/PS1 DS *n* = 5/438; **(C,D)** WT HC *n* = 5/459, APP/PS1 HC *n* = 5/376, WT DS *n* = 5/431, APP/PS1 DS *n* = 5/438. Statistical details are reported in Supplementary Table 1.

Next, we aimed to determine whether there are changes in astrocyte-synapse interaction caused by contextual fear memory. This was studied in both WT and APP/PS1 mice. The animals were sacrificed 4 h post-shock (delayed shock, DS), a timepoint for which we previously showed alterations in proteins expressed in PAPs upon fear memory consolidation (Rao-Ruiz et al., 2015; Sapkota et al., 2022). We found impaired contextual fear memory present in APP/PS1 mice at the age of 3 months (Figure 2A) which is in line with earlier reports by our group (Végh et al., 2014; Kater et al., 2022). To note, we continued our analyses in 4 month old mice.

We established that the number of synapses contacting an astrocyte was unaltered between WT and APP/PS1 mice under control conditions. The numbers observed suggested a reduction in PAP contacts with the post-synapse in control APP/PS1 mice compared to WT, but this difference proved not to be statistically significant. Also, no difference was observed at 4 h following conditioning for APP/PS1 mice, similar to what was observed for WT mice (Figure 2B). Moreover, no alterations were found in pre-synapse size (Figure 2C) or post-synapse size (Figure 2D) in both WT and APP/PS1 mice, and remained unaffected by delayed foot-shock. Together, these findings indicate that synapse structural parameters were not affected when studied at 4 h after contextual fear conditioning, neither it was by early AD pathology.

### 3.3. Contextual fear conditioning-induces retraction of PAPs from the synapse in WT but not in APP/PS1 mice

**FIGURE 3:**
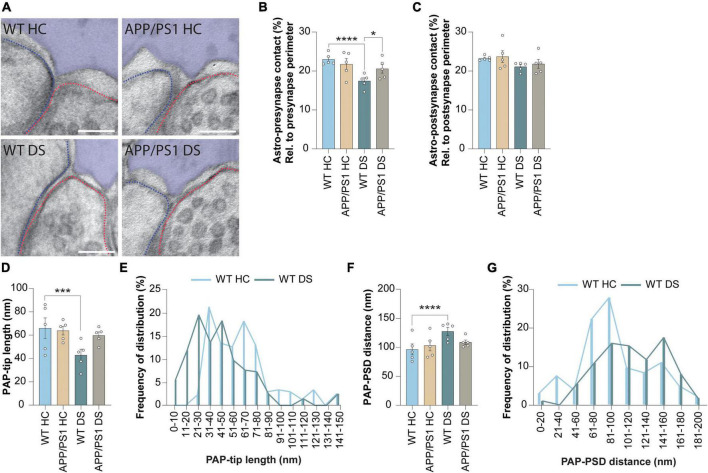
Contextual fear conditioning causes PAP retraction after 4 h in WT but not in APP/PS1 mice. **(A)** Representative images showing WT home cage (HC), APP/PS1 HC, WT delayed shock (DS) and APP/PS1 DS conditions. Astrocyte is pseudo-colored in purple. Outline of the pre-synapse is indicated with the red dashed line, the outline of the post-synapse is indicated by the blue dashed line. Scale bar, 100 nm. **(B)** Shared contact between the astrocyte (Astro) and pre-synapse, relative (rel.) to the pre-synapse perimeter and expressed as percentages (%). **(C)** Shared contact between the astrocyte and post-synapse, relative to the post-synapse perimeter and expressed as percentages. **(D)** Quantification of the length of the PAP tip protruding toward the synaptic cleft. **(E)** Frequency distribution plots of WT HC and WT DS conditions of the data presented in panel **(D)**. **(F)** Quantification of the distance between the PAP-tip and PSD. **(G)** Frequency distribution plots of WT HC and WT DS conditions of the data presented in panel **(F)**. Data is presented as mean ± SEM. *P ≤ 0.05; ***P ≤ 0.001; ****P ≤ 0.0001. **(B)** WT HC *n* = 5/158, APP/PS1 HC *n* = 5/108, WT DS *n* = 5/164, APP/PS1 DS *n* = 5/156; c: WT HC *n* = 5/168, APP/PS1 HC *n* = 5/107, WT DS *n* = 5/173, APP/PS1 DS *n* = 5/169; d + f; WT HC *n* = 5/104, APP/PS1 HC *n* = 5/67, WT DS *n* = 5/94, APP/PS1 DS *n* = 5/98. Statistical details are reported in Supplementary Table 1.

Finally, we studied whether the spatial interaction between the synaptic elements and PAPs was affected 4 h after contextual fear conditioning (Figure 3A). We found no difference in membrane contact between PAP and synapse induced by either genotype or foot-shock (Supplementary Figures 2A, B). However, when the contact between PAP and pre-synapse was measured as a fraction of the total pre-synapse perimeter (Figure 3B), in line with previous studies (Ventura and Harris, 1999; Merino-Serrais et al., 2011; Ostroff et al., 2014), we found a reduction in WT DS mice compared to WT HC. This decreased PAP-pre-synapse contact was not observed in APP/PS1 mice, suggesting that contextual fear conditioning-induced PAP disengagement from the pre-synapse was absent in APP/PS1 mice. Interestingly, the interaction between the PAP and the post-synapse remained unchanged (Figure 3C). Next, we measured whether contextual fear conditioning affected the structure of the PAP-tip. We found that the length of the PAP-tip was reduced in WT DS mice compared to HC mice, while this was unaffected in APP/PS1 mice (Figures 3A, D, E). Furthermore, contextual fear conditioning increased the PAP-PSD distance in WT mice, while not in APP/PS1 mice (Figures 3A, F, G).

Taken together, while the astrocytic synaptic coverage between WT and APP/PS1 is comparable under basal conditions, we reveal that fear memory-induced neuronal activation leads to a reduced PAP-pre-synapse interaction in WT mice and not in APP/PS1. This is accompanied by the shortening of the PAP tip length and increased PAP-PSD distance, again only in WT mice that underwent contextual fear conditioning and not in APP/PS1 mice. Thus, this suggests that activity-induced retraction of the PAPs is a process that occurs during the consolidation of fear memories and that is dysregulated in the early stages of AD pathology.

## 4. Discussion

Our study shows a reliable method to measure *in vivo* astrocyte-synapse interactions by 2D EM. We were able to create a robust dataset with a high number of observations and a low within-group variation, to identify alterations in astrocyte-synapse spatial interaction at nanoscale. We generated high-quality images of PFA fixed brain tissue, which is a great advantage for efficient EM sample preparation. We applied this method here to determine changes during contextual memory consolidation. We confirmed our previous observation that retraction of astrocyte processes from the synapse is implicated in the formation of fear memory (Badia-Soteras et al., 2022). Here, we built on this data and show that fear memory-induced PAP retraction from hippocampal synapses is absent at the early stages of AD.

It has to be noted that the effect sizes of the contact category data we report are possibly an underestimation because of two reasons: First, we restricted our analysis to the specific 2D-plane that was included and therefore we could have missed out on synapses that are in contact with an astrocyte, while not observed in the plane imaged for analysis. One study performing 3D analysis on synapses in the rat hippocampus CA1 determined that 57% of the synapses is in contact with an astrocyte (Ventura and Harris, 1999), whereas we observed this for 48% of the synapses. Other techniques such as super resolution STED microscopy (Arizono et al., 2020) and correlative confocal combined with EM (Aten et al., 2022) reported 55 and 86%, respectively. 3D-reconstructions are often used to study the stellate morphology of astrocytes (Aten et al., 2022). Although 3D analysis better represents single astrocyte morphology, it is time demanding to reconstruct multiple astrocytes per sample. Therefore, such studies included no more than four samples per group, with per group mostly around 10 cells being reconstructed (Ventura and Harris, 1999; Mathiisen et al., 2010; Witcher et al., 2010; Ostroff et al., 2014; Bellesi et al., 2015). Using our 2D-method we are able to include>100 synapses per sample, and five samples per condition, and we argue that in our study this was required to get to significance as we observed a high within sample variation (Supplementary Figure 3). Second, our analysis did not differentiate between synapses that are actively involved in the formation of memory vs. those that are not. It has been reported that 20% of all neurons in the CA1 are activated 2 days after contextual fear conditioning (Tayler et al., 2013), meaning that there is a high chance that we included synapses derived from neurons that are not involved in memory consolidation after shock. This implicates that future studies using approaches that image only synapses of memory engram neurons are expected to find even stronger effects in plasticity of astroglial synapse contact during memory consolidation.

The reduced astrocyte-synapse contact we described was pre-dominantly from the presynaptic terminal, given that astrocyte-post-synapse contact was unaltered. Accordingly, it is thought that PAPs form stable contacts with dendritic spines (Arizono et al., 2020) to support morphogenesis (Murai et al., 2003) and synaptic function (Filosa et al., 2009) and therefore, astrocytes that exhibit shorter PAPs are most likely to disengage from the pre-synapse rather than the post-synapse. In addition, we observed a fear memory-induced shortening of the PAP tip, and consequently a decrease in the proximity of the PAP to the synaptic cleft. This is in line with our earlier study showing a transient retraction of the PAP from the synaptic cleft during the formation and consolidation of fear memory, which causes increased glutamate spill over and enhanced fear memory expression (Badia-Soteras et al., 2022). We should note here as a limitation of our study design that we did not exclude the effect of stress induced by the novel context on our data. Although we demonstrated before that the shock itself does not lead to an additional effect (Badia-Soteras et al., 2022), we should have used non-shocked but conditioning chamber exposed mice as a control instead of HC mice. In line with our data, Connexin-30 deletion causes excessive growth of the PAP tip toward the synaptic cleft, thereby decreasing synaptic glutamate levels and reducing contextual fear memory expression (Pannasch et al., 2014). Importantly, here we observed that the activity-induced retraction of PAPs is absent in APP/PS1 mice with a deficit in fear memory. Thus, optimal astrocyte PAP opposition to the synapse seems critical for synaptic activity and memory processing.

The molecular mechanism underlying the growth or retraction of the PAP tip remains to be determined, but may involve actin binding proteins, such as Ezrin (Badia-Soteras et al., 2022), Cofilin-1 (Henneberger et al., 2020) or Profilin-1 (Molotkov et al., 2013) and activity-induced translation of such proteins locally in the PAPs (Sapkota et al., 2022). Importantly, these molecular processes seem to be impaired in APP/PS1 mice which do not show PAP retraction upon neuronal stimulation. This may be explained by the fact that there is impaired synaptic activity upon DS stimulation in the APP/PS1 brain, however this remains unclear and needs to be determined. An intriguing possibility is that astrocyte reactivity, which is likely to be present in the AD mouse model at this stage (Zhu et al., 2017; Ceyzériat et al., 2018), may interfere with the structural remodeling of PAPs. Pathologically activated astrocytes display a hypertrophy of the processes (Rose et al., 1976) and this is likely to hamper the protrusion of PAPs into the synaptic cleft. We recently showed that the lack of PAP retraction prevents an increase in the perisynaptic space and subsequent glutamate spill over (Badia-Soteras et al., 2022). Whether the uptake or release of other gliotransmitters is affected, either by fear memory or by AD pathology, remains to be determined. Taking together the above data suggest a key role for astrocytes in the underlying mechanism of cognitive impairment in AD.

## 5. Conclusion

In this study we were able to generate a robust dataset with low variability and a high number of observations on astrocyte-synapse interactions measured on nanoscale by 2D EM on PFA fixed brain tissue. Good preservation of morphology was obtained in PFA-fixed tissue cryo-protected, frozen and kept at −80°C before cryostat sectioning and dissection of samples for further processing for EM. This provides great experimental flexibility, accuracy and efficiency of sampling. Using this method, we show that fear conditioning results in the retraction of PAPs from excitatory hippocampal synapses that coincides with contextual fear memory learning, a mechanism we find absent in AD model mice. These findings support the notion that changes in astrocyte-synapse coverage contribute to memory processing.

## Data availability statement

The original contributions presented in this study are included in the article/Supplementary material, further inquiries can be directed to the corresponding author.

## Ethics statement

This animal study was reviewed and approved by the Animal Ethical Care Committee (DEC) of the Vrije Universiteit Amsterdam.

## Author contributions

MK performed the behavioral experiments, EM imaging, and data analysis and statistic. AB-S assisted in behavioral experiments and animal perfusions. JW, MV, and AS were involved in study design. MV and AS supervised the project and secured funding. MK and MV wrote the manuscript with input from all other authors. All authors contributed to the article and approved the submitted version.

## Supplementary material

Supplementary Figure 1 Astrocytes have shorter physical contact with the post-synaptic membrane in comparison with the presynaptic membrane. Data is presented as mean ± SEM. ^****^*P* ≤ 0.0001. Pre-synapse *n* = 5/158, post-synapse *n* = 5/169. Statistical details are reported in Supplementary Table 1.

Supplementary Figure 2 Absolute contact of astrocyte with pre-synaptic or post-synaptic membrane in WT and APP/PS1 mice. **(A)** Shared contact between the astrocyte (Astro) membrane and pre-synaptic membrane, measured in nm. **(B)** Shared contact between the astrocyte membrane and post-synaptic membrane, measured in nm. Data is presented as mean ± SEM. **(B)** WT HC *n* = 5/158, APP/PS1 HC *n* = 5/108, WT DS *n* = 5/164, APP/PS1 DS *n* = 5/156; **(C)** WT HC *n* = 5/168, APP/PS1 HC *n* = 5/107, WT DS *n* = 5/173, APP/PS1 DS *n* = 5/169. Statistical details are reported in Supplementary Table 1.

Supplementary Figure 3 Example images showing the range of variation for astrocyte-synapse structural parameters. **(A)** Pre-synapse perimeter ranging from 1,000 nm till 2,500 nm. **(B)** PAP/PSD distance ranging from 40 nm till 100 nm. **(C)** PAP-tip length ranging from 20 nm till 80 nm. Scale 200 nm. Red dashed line is the pre-synapse perimeter, blue dashed line is the post-synapse perimeter. Astrocytes are pseudo colored in purple.
