# Supplementary figures and images for: Electron microscopy analysis of astrocyte-synapse interactions shows altered dynamics in an Alzheimer’s disease mouse model
Source: Front Cell Neurosci. 2023 Jan 26;17:1085690. doi: 10.3389/fncel.2023.1085690 (PMC9908992; doi:10.3389/fncel.2023.1085690)

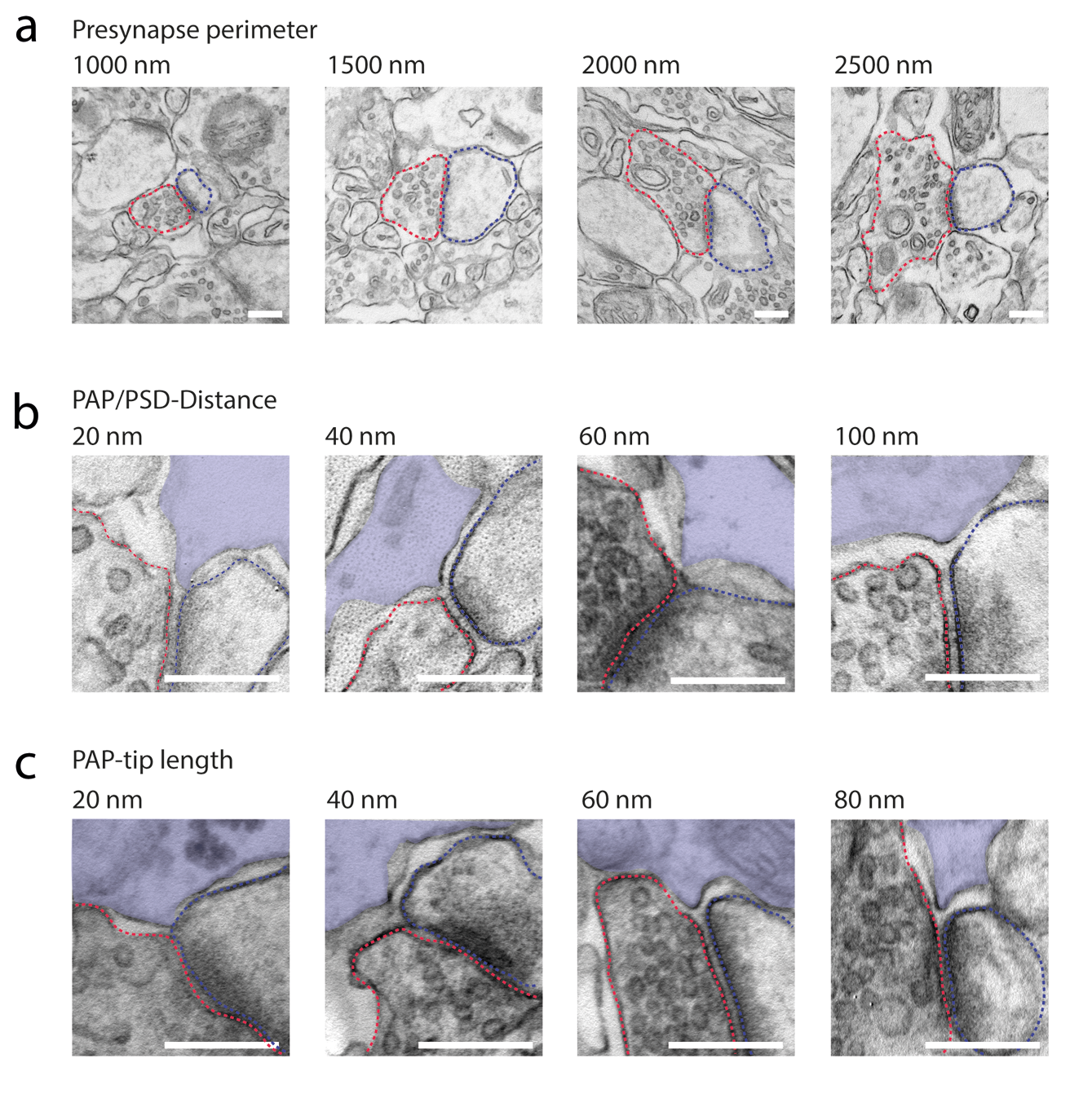

Supplement: Supplementary Figure 3 — Example images showing the range of variation for astrocyte-synapse structural parameters. (A) Pre-synapse perimeter ranging from 1,000 nm till 2,500 nm. (B) PAP/PSD distance ranging from 40 nm till 100 nm. (C) PAP-tip length ranging from 20 nm till 80 nm. Scale 200 nm. Red dashed line is the pre-synapse perimeter, blue dashed line is the post-synapse perimeter. Astrocytes are pseudo colored in purple. [file Image_3.tif]
